# A typical bilateral *Toxoplasma *retinochoroiditis in a bone marrow transplant patient with negative serum titers

**DOI:** 10.1186/1869-5760-3-23

**Published:** 2013-01-28

**Authors:** Albert Hazan, Rakesh M Patel, David Levinson, Umar Mian, David C Gritz

**Affiliations:** 1 Albert Einstein College of Medicine, Bronx, NY, 10467, USA; 2 Department of Ophthalmology and Visual Sciences, Montefiore Medical Center and Albert Einstein College of Medicine, Bronx, NY 10467, USA; 3 Department of Epidemiology and Population Health, Montefiore Medical Center and Albert Einstein College of Medicine, Bronx, 111 East 210th St., Bronx, NY 10467, USA

**Keywords:** Intravitreal clindamycin, *Toxoplasma* retinochoroiditis, *Toxoplasma gondii*

## Abstract

**Background:**

*Toxoplasma* retinochoroiditis can have an atypical presentation and be difficult to diagnose in immunocompromised patients. Accurate diagnosis and appropriate treatment is important since the disease can be aggressive in these patients. This paper is a case report with literature review, emphasizing on the diagnosis and treatment of *Toxoplasma* retinochoroiditis.

**Findings:**

A 27-year-old male with chronic myelogenous leukemia with history of bone marrow transplantation presented with floaters in his right eye. Fundus exam showed bilateral, multifocal retinochoroiditis with subsequent development of a mild vitritis. Serum cytomegalovirus and toxoplasmosis antibody titers and syphilis screen were negative. Aqueous polymerase chain reaction (PCR) analysis revealed the presence of *Toxoplasma gondii* DNA OU. Clindamycin (1.0 mg/0.1 mL) was injected bilateral intravitreal OU twice at 4 days apart with subsequent resolution of retinochoroiditis.

**Conclusions:**

When evaluating retinochoroiditis in an immunocompromised patient, one must keep a high index of suspicion for atypical presentations of well-known disease entities. Aqueous and vitreous samples for PCR can be useful in obtaining an accurate diagnosis and therefore provide appropriate management for the patient. Intravitreal clindamycin is an option for treatment in these patients.

## Findings

### Summary statement

An immunocompromised patient presented with floaters and fundus lesions suspicious for infectious retinochoroiditis vs. chronic myelogenous leukemia (CML) relapse. Despite negative serum toxoplasmosis titers, an aqueous polymerase chain reaction (PCR) analysis revealed ocular toxoplasmosis bilaterally and the patient responded to therapy. We discuss clinical presentation, diagnostic workup, and various treatment options in the management of *Toxoplasma* retinochoroiditis. All research for this brief report was done with appropriate ethical approval from the Albert Einstein College of Medicine Institutional Review Board and informed consent was obtained from the subject.

### Case description

**Figure 1:**
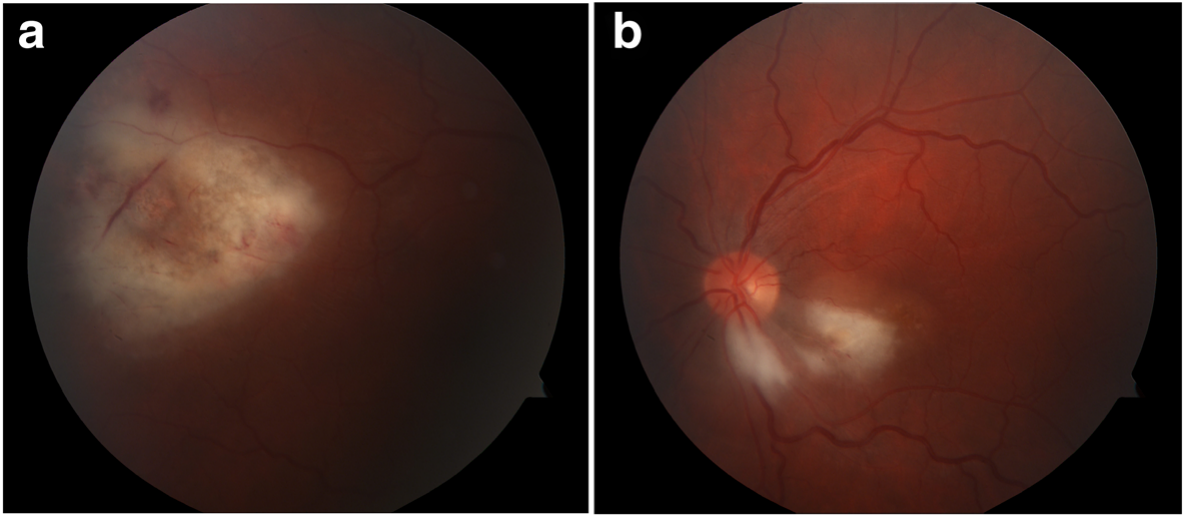
**Fundus examination of the right eye. **Fluffy white retinal infiltrates with mild hemorrhage superotemporal to macula in right eye (**a**) and peripapillary infiltrate in left eye (**b**).

A 27-year-old male with history of CML on remission since treatment with imatinib, chemoradiation, and allogenic bone marrow transplantation 1 year prior to consultation and with active and recurrent acyclovir-resistant herpes simplex stomatosis presented with a 2-day history of floaters in his right eye. Patient’s medications included several immunosuppressive agents such as tacrolimus, sirolimus, and mycophenolate along with the prophylactic medications, atovaquone, valacyclovir, and ciprofloxacin. Visual acuity was 20/30 +3 and 20/100 in the right and left eyes, respectively. Fundus examination of the right eye revealed irregular, fluffy, white subretinal and intraretinal lesions with pigmentary changes in the superotemporal arcade (Figure 1a). In the left eye, a smaller similar lesion was noted just inferotemporally to the optic nerve with adjacent retinal edema involving the macula. No vitritis was noted bilaterally. Differential diagnosis included viral retinochoroiditis (cytomegalovirus (CMV), herpes simplex virus (HSV), and varicella zoster virus (VZV)) vs. CML relapse vs. atypical toxoplasmosis (Figure 1b).

Serum IgM and IgG titers for CMV and toxoplasmosis were negative in addition to syphilis screening being negative. Cerebrospinal fluid (CSF) analysis was negative for CMV, HSV, toxoplasmosis via PCR, and bacterial growth. CSF analysis also showed atypical cells with no blasts indicating no evidence of CML relapse. MRI brain/orbits showed no abnormalities. Intravenous cidofovir had recently been started for HSV stomatitis, and the patient was initially observed on this treatment.

**Figure 2:**
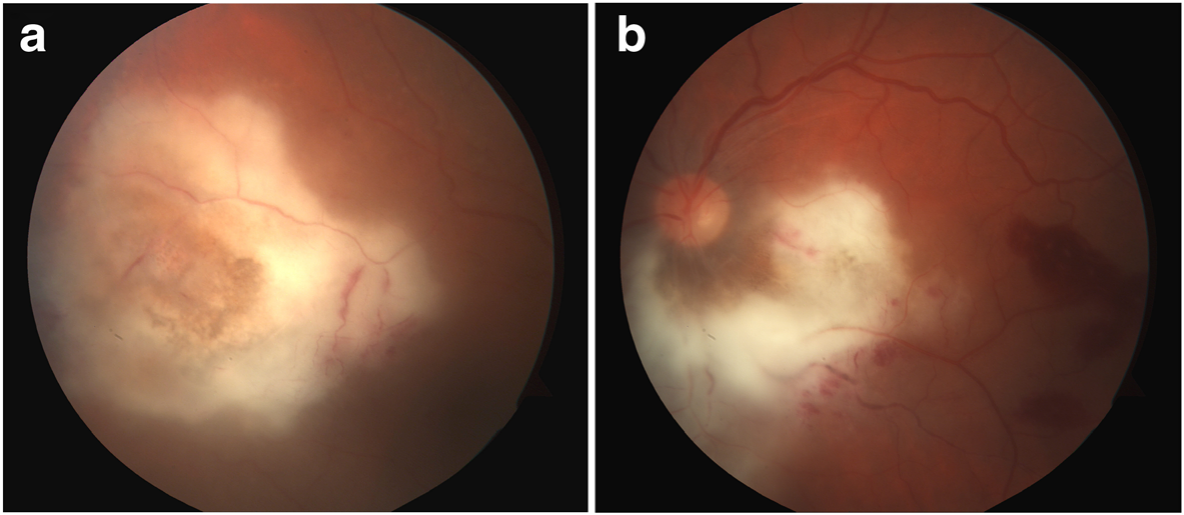
**Progression of the retinal lesions with a mild vitritis. **Increased density and size of infiltrates in the right eye (**a**) enlarging the peripapillary infiltrate with new adjacent retinal hemorrhages in the left eye (**b**). There were suggestions of subretinal inflammation clinically, but unfortunately, not visible in photos.

Two weeks after presentation, visual acuity decreased to 20/70 OD and 20/400 OS. Examination revealed progression of the retinal lesions with a mild vitritis (Figure 2). The clinical picture was highly suspicious for CMV retinitis; however, the negative serum CMV PCR and progression of lesions on cidofovir raised serious doubt. An anterior chamber paracentesis was performed, and the aqueous humor was sent for PCR for CMV, HSV, VZV, Epstein-Barr virus (EBV), and toxoplasmosis. A more aggressive treatment of the CMV retinitis was also pursued with bilateral intravitreal injections of foscarnet in addition to intravenous route.

**Figure 3:**
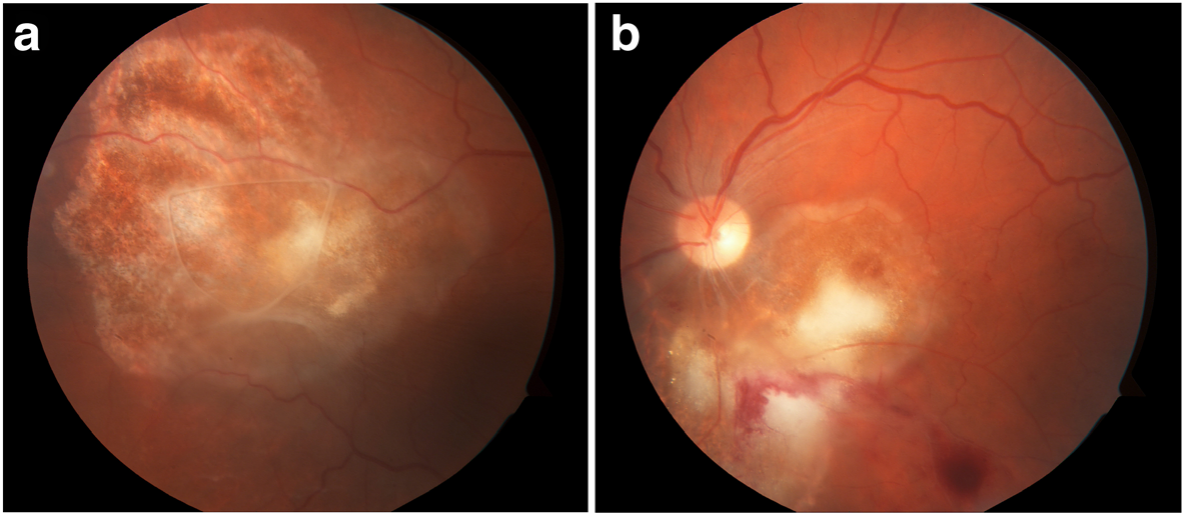
Note granular appearance of lesions with gliosis indicating resolving retinochoroiditis, in both the right eye (a) and left eye (b).

Five days later, quantitative PCR analysis revealed 3,400 DNA copies/ml OD and 2,800 DNA copies/ml OS of *Toxoplasma gondii* and negative PCR for HSV, VZV, EBV, and CMV OU. A diagnosis of *Toxoplasma* retinochorioditis was made. Clindamycin (1.0 mg/0.1 mL) was injected intravitreally OU twice at 4 days apart. Over the next month, the active areas of retinochoroiditis regressed followed by gliosis of the lesions with pigmentary changes (Figure 3a,b). The vision subsequently improved to 20/50 OD and 20/100 OS. Patient was also started on trimethoprim-sulfamethoxazole to prevent future reactivation of the disease.

## Discussion

This case report describes a patient with *Toxoplasma* retinochoroiditis masquerading as a viral infection in a patient with chronic myelogenous leukemia. Initial clinical examination and laboratory values lead to a presumptive diagnosis of a viral retinochorioditis, and treatment with both intravitreal and systemic foscarnet was initiated in addition to oral cidofovir. Disease progression was noted with the increasing size of the retinal lesions. Diagnostic aqueous tap sent for PCR analysis confirmed the diagnosis of toxoplasmosis and the patient was successfully treated with intravitreal injections of clindamycin.

It may be difficult to clinically distinguish toxoplasmosis infection among the different causes of infectious retinochoroiditis; however, such a distinction has important therapeutic implications. It has been described that when contrasted to CMV retinochoroiditis, retinal lesions of toxoplasmosis are thicker and more densely opaque with smooth and non-granular lesion borders which are often associated with a chorioretinal scar [1]. Subretinal fluid in association with retinochoroiditis is more commonly seen in toxoplasmosis [2]. Prominent inflammatory reactions, anterior and posterior, are a hallmark of *Toxoplasma* retinochoroiditis [1]. However, in immunocompromised patients, the inflammatory reaction may not be as robust, thereby diminishing the sensitivity of this difference. Additionally, one of the most interesting aspects of this case was the negative serum and CSF toxoplamosis serology, which swayed the diagnosis away, until the results of the aqueous PCR were received. Again, the negative serology may have been due to the poor immune function of this patient with a history of CML s/p bone marrow transplantation. Furthermore, the rapid progression of the disease process in this patient is atypical for toxoplasmosis. Toxoplasmosis may present bilaterally with rapid progression, without impressive inflammation and a negative serum serology, in immunocompromised patients. Thus, a high index of suspicion is needed to make the diagnosis of *Toxoplasma* retinochoroiditis in such patients.

Treatment for this patient was with two sets of bilateral intravitreal injections of clindamycin (1.0 mg/0.1 mL). It has been shown that intravitreal clindamycin plus dexamethasone has similar outcomes to classic therapy (pyrimethamine, sulfadiazine, and prednisolone) for *Toxoplasma* retinochoroiditis in terms of the ability to reduce lesion size and vitreal inflammation as well as improve visual acuity [3,4]. Because of issues with follow-up, the two injections were given 4 days apart. The patient responded to only two injections so well, that he was simply observed at that point.

Oral clindamycin has also been used in the treatment of *Toxoplasma* retinochoroiditis. Some use it as part of a quadruple therapy in immunocompetent patients, which consists of classic therapy plus oral clindamycin [5]. It has also been shown that oral clindamycin alone is effective in treating *Toxoplasma* retinochoroiditis with minimal side effects [6].

Intravitreal administration of clindamycin has advantages over classic therapy due to its reduced side effect profile. Intravitreal injections of clindamycin deliver a high concentration of the medicine to the intraocular tissues while reducing systemic absorption and unwanted systemic side effects. Additionally, the clindamycin’s good intracellular penetration makes it a good antimicrobial choice for the intracellular *T. gondii.* Several studies have shown clindamycin to be non-toxic to the retina [3]. Nevertheless, the risks of an intravitreal injection itself do remain including endophthalmitis and retinal detachment [7,8]. Furthermore, intravitreal clindamycin will only treat the ophthalmic disease, but it will not treat nor prevent systemic reactivation of toxoplasmosis.

Another appropriate treatment for ocular toxoplasmosis is trimethoprim-sulfamethoxazole (TMP-SMX). It has been shown that TMP-SMX has similar efficacy in terms of reduction in the retinal lesion size and improvement in visual acuity and has preferred side effect profile compared to pyrimethamine and sulfadiazine [9]. Given as an oral medication, it will provide prophylaxis for systemic reactivation of the disease as well.

Multiple different regimens have been shown to be effective in treating toxoplasmosis retinochoroiditis in immunocompromised patients, none of which proving to be superior. In contrast to immunocompromised patients in whom treatment is crucial, it is not established that immunocompetent patients require therapy.

## Conclusions

Toxoplasmosis should be included in the differential diagnosis of a retinochoroiditis in an immunocompromised patient even if it presents without the typical inflammatory response, an adjacent chorioretinal scar, typical retinitis, or is bilaterally active. If the CSF and serum serologies are negative, this should not sway the diagnosis away from toxoplasmosis in the immunocompromised patients. One should consider an aqueous tap for PCR analysis to aid in the diagnosis of the disease process. Once the diagnosis is confirmed, appropriate therapy may be promptly instituted in hopes of preserving the maximal vision.

## Competing interests

The authors declare that they have no competing interests.

## Authors’ contributions

AH drafted the manuscript and compiled a literature search. RP, DL, UM, and DG all played a role in clinical diagnosis and treatment decision making as well as editing the manuscript. All authors read and approved the final manuscript.
